# Comment on “Investigating the necessity of preoperative coronary angiography for infection-related cardiac implantable electronic device explantation”

**DOI:** 10.1016/j.ahjo.2026.100902

**Published:** 2026-09-16

**Authors:** Saurav Gupta, Priyadarshini S. Nayak

**Affiliations:** a Dr. Ram Manohar Lohia Institute of Medical Sciences, Vibhuti Khand, Gomti Nagar, Lucknow, Uttar Pradesh, India

**Keywords:** Coronary angiography, Cardiac implantable devices, Device explantation, Device infection, Preoperative assessment, Surgical management

Dear Editor,

We read with great interest the study by Caldonazo et al. [1], that evaluated preoperative coronary angiography before CIED explantation.

## 1 Baseline imbalance and the interpretation of equivalent outcomes

The angiography group had significantly more lead vegetation, more two-vessel coronary disease, and a higher proportion of leads in place beyond one year than the non-angiography group. These differences indicate that a more anatomically complex and higher-risk cohort was selected for coronary angiography [2]. However, the 30-day mortality and complication rates were compared with unadjusted bivariate tests alone, and the EuroSCORE II reported as similar between groups does not capture the lead vegetation burden or duration of device implantation. Equivalent crude outcomes between a higher-risk angiography-assessed group and a lower-risk unassessed group are compatible with angiography, providing a real protective benefit that offsets the underlying risk difference, and angiography is not only unnecessary. Without a model that accounts for these documented baseline differences, the conclusion that coronary angiography does not influence outcomes cannot be distinguished from the alternative explanation that it neutralizes a risk gradient that would otherwise have separated the groups.

## 2 What angiography actually changed at the operating table

No coronary artery bypass grafting was performed after any of the four surgical conversions, and every documented reason for conversion was lead-related rather than coronary artery bypass grafting. The manuscript reports whether patients died or developed complications but not whether angiography findings altered anesthetic planning, perfusion strategy, or surgeon-reported confidence proceeding with sternotomy in a comorbid population [3]. Because no patient in either arm ultimately required bypass, this cohort by construction cannot show what angiography prevents; a null result on hard outcomes in a scenario where the feared complication never materialized in either group does not establish that angiography adds nothing when such a complication occurs.

## 3 An undifferentiated comparator group

Patients who did not undergo angiography were selected based on the surgeon's judgment, and per the methods, sometimes underwent unspecified alternative diagnostic strategies [4]. The manuscript does not report how many of the 167 non-angiography patients received any coronary assessment at all versus none. Since the discussion recommends coronary computed tomography as a substitute for invasive angiography, knowing whether any non-angiography patients already had equivalent non-invasive imaging and whether their outcomes differed from those with no coronary assessment is necessary before treating the entire non-angiography group as evidence that omitting coronary assessment altogether is safe.

## 4 Conclusion

The comparison itself and the low observed rate of bypass in this population are genuinely useful observations for a question with little existing literature. Reporting outcomes adjusted for the documented baseline differences, describing what alternative assessment the non-angiography patients received, and capturing whether angiography changed intraoperative decisions rather than only whether patients survived would let readers judge whether coronary angiography can be omitted, or whether its apparent lack of effect reflects a cohort and a set of conversions in which it was rarely put to a genuine test.

## CRediT authorship contribution statement

**Saurav Gupta:** Conceptualization, Data curation, Formal analysis. **Priyadarshini S. Nayak:** Visualization, Writing – original draft, Writing – review & editing.

## Ethical approval

Not applicable (commentary on previously published research).

## Declaration of Generative AI and AI-assisted technologies in the writing process

Generative AI tools were used solely for language refinement and formatting assistance. All scientific interpretation, critique, and conceptual analysis were independently developed by the authors.

## Funding

None.

## Declaration of competing interest

The authors declare no conflicts of interest.

## Data Availability

No new data were generated or analysed in this work.

## References

[1] Caldonazo T., Scheler H., Fischer J., Kirov H., Mukharyamov M., Gräger S., Runkel A., Reinartz S., Diab M., Doenst T. (2026). Investigating the necessity of preoperative coronary angiography for infection-related cardiac implantable electronic device explantation. Am. Heart J. Plus.

[2] Lebowitz M., Modanwal G., Dhamdhere R., Mutha P., De Cecco C., van Assen M., Madabhushi A. (2025). Abstract 4365988: AI-informed coronary artery tortuosity index (CArTI) from cardiac CT angiography predicts 5-year cardiovascular risk. Circulation.

[3] Li M., McGrail D., Mahmood F., Bose R. (2025). Pre-operative cardiac assessment for coronary artery disease: from symptoms to angiography. J. Cardiothorac. Vasc. Anesth..

[4] Iliopoulos J. (2024).

